# The effects of hormonal changes on sperm DNA integrity in oligoasthenoteratospermia individuals: A case-control study

**DOI:** 10.18502/ijrm.v20i12.12560

**Published:** 2023-01-09

**Authors:** Zeinab Bahrami, Neda Daeifarshbaf, Fatemehsadat Amjadi, Reza Aflatoonian

**Affiliations:** ^1^Department of Biology, Science and Research Branch, Islamic Azad University, Tehran, Iran.; ^2^Laleh IVF Clinic, Laleh Hospital, Tehran, Iran.; ^3^Department of Anatomy, School of Medicine, Iran University of Medical Sciences, Tehran, Iran.; ^4^Infertility Center, Imam Khomeini Hospital, Mazandaran University of Medical Sciences, Sari, Iran.; ^5^Department of Endocrinology and Female Infertility at Reproductive Biomedicine Research Center, Royan Institute for Reproductive Biomedicine, ACECR, Tehran, Iran.

**Keywords:** DNA fragmentation, Oligospermia, Asthenospermia, Teratospermia, Hormones.

## Abstract

**Background:**

Hormonal imbalance is one of the important etiological factors for Oligoasthenoteratospermias (OAT).

**Objective:**

This study aimed to evaluate the effects of hormonal changes including prolactin, TSH, testosterone, luteinizing hormone, follicle-stimulating hormone, and anti-Mullerian hormone on sperm DNA fragmentation in normal men compared with OAT to design a clinical algorithm for the comprehensive study of male factor infertilities.

**Materials and Methods:**

We consecutively selected 60 candidates referred to the infertility clinic to collect the semen and blood samples. Then, a terminal deoxynucleotidyl transferase dUTP nick end labeling test was performed to evaluate the sperm DNA fragmentation index (DFI). After semen analysis and DFI checking, they were classified into 4 groups consisting of normospermia and OAT men each with or without increased DFI. Hormone parameters were analyzed using enzyme-linked immunoassay.

**Results:**

Follicle-stimulating hormone and luteinizing hormone levels showed positive correlations with DFI in a significant way (p ≤ 0.01), while testosterone and thyroid-stimulating hormone were associated with sperm concentration. Prolactin and anti-Mullerian hormone levels significantly correlated (p ≤ 0.01) with sperm concentration and DFI value simultaneously.

**Conclusion:**

Decreased and increased levels of serum hormones could adversely affect semen profile and sperm DNA integrity which lead to severe male infertility. Although we investigated the effects of the main hormones related to male infertility on DNA damage, the role of these hormones on the fertilization rate and embryo quality needs to be evaluated in further studies.

## 1. Introduction 

Infertility affects approximately 15% of couples attempting pregnancy. Also, the male factor is responsible for about half of these cases (1). It is well-established that most of the male infertility problems associated with qualitative and quantitative defects of spermatogenesis, lead to sexual and fertility dysfunction (1, 2). Abnormalities of the seminal parameters of oligoasthenoteratospermia (OAT) are important causes and the main contributory reasons for male infertility (3). Apart from the variety of known factors for OAT such as age, systemic diseases, varicocele, infection, cryptorchidism, testicular trauma, obstructions, immunological factors, endocrine disorders, and idiopathic factors, one of the critical underlying etiologies is hormonal imbalance (4-6).

There are growing concerns that disturbance in hormone levels results in spermatogenesis dysfunction and male infertility. Hormone deficiency in germ cell microenvironments has been indicated as the key reason for immature sperm increase which could adversely affect sperm survival and function and may lead to the authors answering to reviewer's comments and correcting some of them, which was acceptable (7). Single and double DNA strand breaks are positively related to sub-haploid and sperm late apoptosis (8). Given together, it is concluded that high sperm DNA fragmentation index (DFI) levels could reduce fertility capacity by affecting the quality and quantity of the spermatogenesis process (9).

The process of spermatogenesis is thoroughly dependent on downstream hormones secreted in response to gonadotropin-releasing hormone in the hypothalamus-pituitary-testis axis (10). The normal hormonal function of the testis is modulated by pituitary secretion of follicle-stimulating hormone (FSH), luteinizing hormone (LH), prolactin (PRL), thyroid-stimulating hormone (TSH), testosterone, and anti-Mullerian hormone (AMH) (11, 12). FSH affects Sertoli cells to produce AMH or serves a direct fundamental role in spermatogenesis through stimulation of cell proliferation, differentiation, and control of surrounding cells apoptosis (10, 13). However, LH acts on Leydig cells to promote testosterone secretion and affects spermatogenesis via testosterone/androgen receptors (14). Although many studies have been carried out on the beneficial effects of these hormones on spermatogenesis, there are many controversial findings in different studies (15-18). Therefore, in the current study, we investigated the effects of known hormones on sperm DFI to draw a more reliable conclusion.

In addition to the most studied hormones, it is reported that PRL and thyroid hormone disorders affect sexual dysfunction and impair the female reproductive function, but their effects on the male reproductive system need to be studied in detail (19, 20). However, there is evidence that the cooperation of PRL and gonadotropins along the hypothalamus-pituitary-testis axis and the effects of thyroid hormones on fetal Sertoli cell maturation and Leydig cell differentiation are crucial for normal spermatogenesis (21, 22).

However, much research is still needed in this area. Hence, the novelty of our study was to investigate the effects of PRL and TSH, along with other hormones including FSH, LH, testosterone, and AMH, on sperm DNA integrity of normal men compared with OATs to design a clinical algorithm for the comprehensive study of male factor infertilities.

## 2. Materials and Methods

### Participants

In this case-control study, samples of semen and blood were collected from 60 men (25-45 yr) referred to the infertility clinic for evaluation or treatment of infertility (Infertility Clinic in Laleh hospital, Tehran, Iran), between May 2018 and February 2019. The included men had no history of chronic illness, chemotherapy, radiotherapy, varicocele, and abnormal testicular size. None had used antioxidant or hormonal therapy during the previous 3 months. Since exogenous agents like smoke are important influencing factors on seminal parameters, heavy smokers were excluded from the analysis. 2 groups were included in this study according to semen profiles: an OAT group (n = 30) and a normal control group (n = 30). The control group included men with normal sperm parameters. After semen analysis for the final classification of individuals, DFI checking terminal deoxynucleotidyl transferase dUTP nick end labeling (TUNEL assay) and hormonal analysis (enzyme-linked immunoassay test) were performed in both groups. Therefore, participants were classified into 4 groups, normospermia with DFI < 19.25, normospermia with DFI > 19.25, OAT with DFI < 19.25, and OAT with DFI > 19.25 (23).

### Analysis of seminal parameters

Samples were collected by masturbation without condom or lubricant cream, after at least 2 days of sexual abstinence and were allowed to liquefy for 30 min at 37 C. After liquefaction, semen parameters (concentration, total motility, and morphology) analyses were conducted according to the World Health Organization 2010 and Kruger's criteria (24). 3 independent replicates were processed and evaluated separately for each semen sample. Sperm morphology was analyzed with a Diff-Quick kit (BRED Life Science Technology Inc., China) and classified by the Kruger classification. The individual was considered OAT by the following criteria: sperm count below 15 million/ml, motility under 40%, and morphology below 4%.

### DFI analysis

An In-Situ Cell Death Detection kit (Roche Diagnostics GmbH) was used to carry out the TUNEL assay and analysis of terminal deoxynucleotide end labeling in sperm-containing DFI. After the preparation of samples based on the manufacturer's protocol, fluorescence signals of the spermatozoa were examined by flow cytometric analysis. For this purpose, about 10,000 events were evaluated with an excitation wavelength of 488 nm on the FACS Calibur flow cytometer (Becton and Dickinson Co.). The signals related to TUNEL-positive cells (green fluorescence) were measured by a 530 ± 30 nm bandpass filter. The positive and negative controls were prepared following the manufacturer's instruction, by incubating with 50 µg/ml DNase I and omitting terminal deoxytransferase (TdT), respectively (25).

### Laboratory analysis

Blood analysis were performed (Serology laboratory of Infertility Clinic in Laleh hospital, Tehran, Iran). The hormone parameters were analyzed in peripheral blood samples collected in the morning from 7:30-9:00 AM, after fasting overnight. The samples were centrifuged for 10 min at 3000 rpm and the serum was immediately frozen at -20 C temperature. All experiments were performed in triplicate. Then, FSH, LH, AMH, testosterone, PRL, and TSH hormones were measured by an enzymatic immunoassay (ELISA, Vidas, France, Marcy). The normal adult ranges for our laboratory are FSH 2-10 IU/L, LH 1-8 IU/L, AMH 1-6 ng/mL, testosterone 2-8 ng/dL, PRL 2.5-17 ng/mL, and TSH 0.4-4 mIU/L.

### Ethical considerations

All procedures were as per the ethical standards of the responsible committee on human experimentation (institutional and national) and with the Helsinki Declaration of 1964 and its later amendments (Code: IR.IUMS.REC.1401.462). Informed consent were obtained before the sample collection from all participants. This study was approved by the Ethics Committee.

### Statistical analysis 

Statistical Package for the Social Sciences software (SPSS, version 23; IBM Corp) was used to carry out statistical analysis. One-way ANOVA followed by Tukey's test was performed to compare quantitative variables between groups. All data were presented as mean ± SEM and the p-value < 0.05 was considered to indicate a statistically significant difference.

## 3. Results

### Sperm parameters and DFI

**Figure 1:**
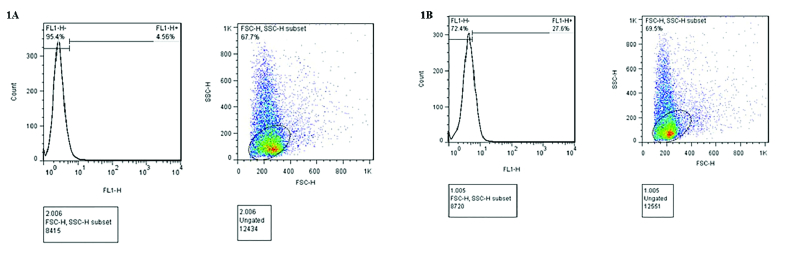
Terminal deoxynucleotidyl transferase dUTP nick end labeling (TUNEL) assay. (A) normospermia sample with a low level (4.56%) of TUNEL-positive sperms, (B) normospermia sample with a high proportion (27.6%) of TUNEL-positive sperms*, *FSC: Forward-angle light scatters, SSC: Side-angle light scatter, FL1-H: Fluorescence intensity.

The results of semen analysis and the TUNEL test are demonstrated in table I. Semen volume, sperm concentration, motility, and morphology were analyzed according to the World Health Organization (2010) and Kruger's criteria. All semen characteristics were significantly different between the control (normospermia) and case (OAT) groups (Table I). Individuals in groups 2 and 4 had statistically significant higher in the percent of DFI compared to the groups 1 and 3 (p = 0.001). Figure 1 shows the result of the TUNEL test. The DFI of sperm was acceptable according to the TUNEL assay with a cutoff point of less than 19.25% (Figure 1A). Specimens with more than 19.25% TUNEL-positive sperms were considered high DFI (Figure 1B).

### Hormonal levels and DFI

**Figure 2:**
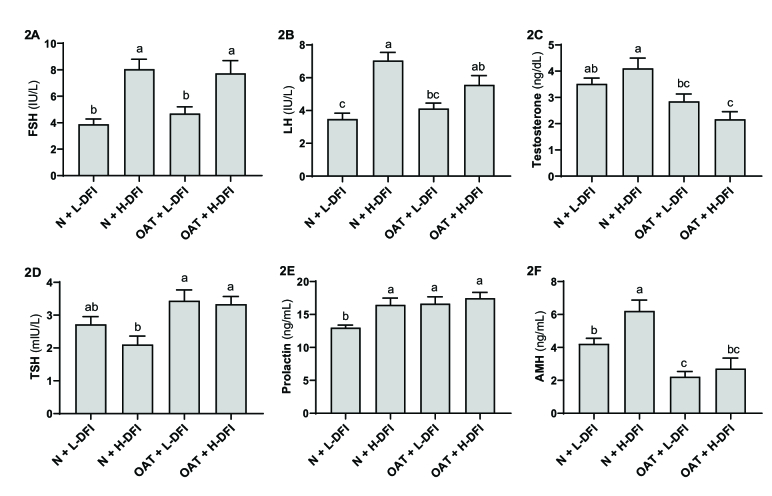
Levels of A) Follicle-stimulating hormone, B) Luteinizing hormone, C) Testosterone hormone, D) Thyroid-stimulating hormone, E) Prolactin hormone, and F) Anti-mullerian hormone with DFI < 19.25, normospermia with DFI > 19.25, oligoasthenoteratospermia with DFI < 19.25, and oligoasthenoteratospermia with DFI > 19.25 groups. The results are shown based on Mean ± SEM. Each number of groups is 15. N+L-DFI: Normal sperm + low DFI, N+H-DFI: Normal sperm + high DFI, OAT+L-DFI: Oligoasthenoteratospermia + low DFI, OAT+H-DFI: Oligoasthenoteratospermia + high DFI. † One-way ANOVA followed by Tukey's test was performed for statistical analysis. a-b groups followed by the same letter are not significantly different at the 0.05 level.

Figure 2 shows the mean concentration of hormones in 4 groups. As depicted in figure 2A, FSH levels in normospermia men with low DFI (control group) decreased significantly when compared to the normospermia and OAT groups with high DFI (p < 0.01). Moreover, FSH concentrations in the OAT group with high DFI were noticeably higher than the OAT group with low DFI (p < 0.01).

LH levels was significantly higher in OAT and normospermia groups with high DFI compared to the control group (p < 0.01, Figure 2B). The level of testosterone (Figure 2C) in normospermia groups reduced significantly in comparison to the OATs with high DFI levels (p < 0.01). Inversely, as shown in figure 2D, the average range of TSH in both OAT groups were significantly higher than normospermia with high DFI (p < 0.01). Although there was a relationship between the last 2 hormones and the sperm concentration, this correlation was positive for testosterone and negative for TSH. As shown in figure 2E, PRL levels in the control group with normal spermogram and low DFI value were remarkably lower than all other 3 experimental groups (p < 0.01). Based on our results, PRL level correlates significantly (p ≤ 0.01) with low sperm concentration and high DFI value simultaneously. AMH levels was remarkably higher in the normospermia group with high levels of DFI compared to the control group (Figure 2F). Additionally, AMH levels were significantly lower in the blood samples of individuals with OAT regardless of sperm DFI level in comparison to the normospermia groups. According to our analysis, increased and decreased levels of AMH had a significant relationship with DFI value and sperm concentration (p ≤ 0.01).

**Table 1:** Relationship between sperm parameters and DFI

**Sperm parameters**	**Groups**	**P-value**	**F (3, 56)†**
	**Normal sperm (DFI < 19.25)**	**Normal sperm (DFI > 19.25)**	**Oligoasthenoteratospermia (DFI < 19.25)**	**Oligoasthenoteratospermia (DFI > 19.25)**		
**Concentration**	74.33 ± 42.58a	65.73 ± 32.46a	5.47 ± 3.44b	6.67 ± 4.84b	< 0.001	28.46
**Total motility**	57.13 ± 22.06a	49.07 ± 19.17a	24.73 ± 16.57b	25.13 ± 20.56b	< 0.001	10.65
**Morphology**	5.13 ± 0.74a	4.13 ± 1.64a	2.47 ± 0.52b	1.87 ± 1.41b	< 0.001	24.62
**DFI**	13.20 ± 4.96b	35.80 ± 3.34a	16.00 ± 2.42b	32.93 ± 6.24a	< 0.001	99.00

Results are shown based on Mean ± SEM. † One-way ANOVA followed by Tukey's test. ${}^{\text{a-b}}$ Indicates being meaningful (p ≤ 0.05). Group 1: Normal sperm and DNA fragmentation index (DFI) < 19.25, group 2: Normal sperm and DFI > 19.25, group 3: Oligoasthenoteratospermia and DFI < 19.25, group 4: Oligoasthenoteratospermia and DFI > 19.25. a-b: Groups followed by the same letter are not significantly different at the 0.05 level

## 4. Discussion 

Our result showed that these hormones have comprehensive effects on male fertility potential and could affect sperm DNA integrity along with semen parameters. OAT, as the most challenging condition in infertility clinics, is associated with the abnormal spermatogenic process, which could be caused by hormonal imbalance (26). Current study on OATs revealed that FSH and LH levels could be indicative of DFI levels, while testosterone and TSH are associated with sperm concentration.

To the best of our knowledge, the present study was the first to examine the effects of PRL and AMH levels on sperm DFI in OAT individuals. Gonadotropin and testosterone concentrations play significant roles in the maintenance of normal spermatogenesis. Furthermore, any alteration in their serum levels could cause abnormal semen profile, sperm DFI, and finally fertility problems (27). Accordingly, our results demonstrated that FSH and LH concentrations were elevated in the groups with increased DFI values.

In the current study, the average concentration of gonadotropins increased significantly by increasing the DFI value, but all levels were in the normal range. Based on our findings, FSH levels were significant determinants of the DFI value which had previously been proven (28). On the other hand, several studies demonstrated the positive effects of FSH pretreatment in the improvement of sperm DNA integrity, but the results are controversial in OATs (29-31). Since OAT is sometimes associated with higher serum levels of gonadotropins, the advantages of FSH treatment on sperm DFI will be promising when endogenous FSH serum level is below 8 IU/L (26, 32). Taken together, it seems that FSH administration for our individuals with high DFI could not be beneficial due to their FSH levels around 8 IU/L.

Pretreatment of these individuals with gonadotropin might alter the hormonal balance, elevated FSH to upper than the normal range, and deteriorate their clinical condition. Therefore, it lightens the possibility that in the case of increased sperm DFI with higher concentrations of serum FSH ( > 8 IU/L), alternative antioxidant supplementation could be preferred to suppress the apoptosis and preserve fertility potential. Lu evaluated the relationship between reproductive hormones and sperm DFI in 1010 subfertile men. They observed positive correlations between FSH and LH levels and sperm DFI but no relationship was found between testosterone levels and DFI value which supports our results (28). The present study indicated a relationship between testosterone level and seminal parameters. Our findings are partly consistent with those obtained by Trussell, who observed that in men with unexplained infertility, low testosterone level was associated with abnormal sperm morphology and caused lower live birth rates. Contrary to our results, there was no significant correlation between testosterone level and other parameters of semen such as sperm concentration, motility, and semen volume (33). The variation in the results of these studies could be due to the different target groups (OATs vs. unexplained male patients). Besides FSH, LH, and testosterone, the current study has addressed the effect of TSH, PRL, and AMH on DFI and semen profile. According to our results, TSH showed a significant correlation with seminal parameters in a negative way.

A study showed that men with abnormal semen parameters had higher total T3, T4, and lower TSH levels compared to those with normal semen profiles (34). Nikoobakht investigated the effect of hypothyroidism on semen profile and found that a high level of TSH, in hypothyroidism, adversely affects sperm count, motility, and morphology (35). Unlike previous findings and our results, an investigation indicated that high levels of TSH only is associated with reduced sperm morphology but no other parameters (34).

It has been demonstrated that the excess of PRL, like TSH, could reversibly affect male reproduction. Timely diagnosis of hyperprolactinemia in males can prevent irreversible infertility and unnecessary invasive procedures for achieving pregnancies (36). Untreated hyperprolactinemia could affect spermatogenesis and reduce sperm motility and counts (37). Keskin also evaluated the effects of hyperprolactinemia on semen parameters. However, there was no significant association between the PRL level and seminal parameters, but they suggest that PRL concentrations should be analyzed in moderate and severe oligozoospermias with a sperm concentration < 10 million/ml (38). Our findings are of interest, considering the effect of PRL on sperm DFI. According to our results, there is a positive correlation between PRL levels and DFI value, however, further studies are needed, especially in the hyperprolactinemia individuals after complete treatment. The last hormone evaluated in the current research is peptide growth and differentiation factors called AMH which plays an important role in spermatogenesis. AMH is produced by Sertoli cells which may reflect its critical effects in normal testicular function and spermatogenesis (39). In the support of this fact, the role of AMH in the normal spermatogenic process through a positive relationship with semen parameters has been confirmed previously (40). However, perverse to our results, Appasamy had observed no significant correlation between AMH and sperm DFI value (41). To obtain reliable findings and eliminate the inconsistency of results, it is necessary to further examine the functional mechanism of AMH in maintenance of sperm DNA integrity in male infertilities.

## 5. Conclusion

In summary, the present study suggests that the fluctuations in the serum levels of mentioned hormones could adversely affect semen profile and sperm DNA integrity which lead to severe fertility problems such as OAT. Accordingly, our findings demonstrated that FSH and LH levels could be indicative of DFI levels, while testosterone and TSH are associated with sperm concentration. Based on the current results, there were also positive correlations between PRL and AMH levels with sperm DFI value. However, the possible effect of these hormones in male factor infertility, on the fertilization rate and embryo quality, needs to be evaluated.

##  Conflict of Interest

The authors declare that they have no competing interest.
